# Editorial: Mitochondrial disorders and cardiovascular diseases

**DOI:** 10.3389/fphys.2023.1179922

**Published:** 2023-05-18

**Authors:** Quanjiang Zhang, Qiuxia Li, Wenjuan Xing

**Affiliations:** ^1^ Division of Endocrinology, Diabetes and Hypertension, Department of Medicine, David Geffen School of Medicine and UCLA Health, University of California-Los Angeles, Los Angeles, United States; ^2^ Division of Cardiovascular Medicine, Division of Endocrinology and Metabolism, Department of Internal Medicine, Abboud Cardiovascular Research Center, and Fraternal Order of Eagles Diabetes Research Center, Carver College of Medicine, University of Iowa, Iowa City, IA, United States; ^3^ Key Laboratory of Aerospace Medicine, Ministry of Education-School of Aerospace Medicine, Fourth Military Medical University, Xi’an, China

**Keywords:** mitochondrial dysfunction, cardiovascular diseases, mtROS, calcium, mitochondrial dynamics

Mitochondrial dysfunction is defined as a loss of efficiency in the electron transport chain and reductions in the synthesis of high-energy molecules. The manifestation of mitochondrial dysfunction includes a decrease in ATP production, an increase in reactive oxygen species (ROS) generation, mitochondrial swelling, alterations of mitochondrial dynamics and mitophagy, opening of mitochondrial permeability transition pore (mPTP), and concomitant leak of damage-associated molecular patterns (DAMPs), which transform mitochondria from a powerhouse into a death engine. Mitochondrial dysfunction is commonly observed in cardiac and vascular tissues of patients with cardiovascular diseases and animal models of cardiovascular diseases (Ait-Aissa K. et al., 2019; Ait-Aissa K. et al., 2019). Of note, the above-mentioned mitochondrial alterations, to a certain extent, precede and accompany the development of various cardiovascular diseases. When these dysfunctional processes are reversed or blocked, cardiovascular diseases may be alleviated. Therefore, mitochondrial dysfunction is considered a contributing factor in various cardiovascular diseases. The articles on the Research Topic *“Mitochondrial disorders and cardiovascular diseases”* provides some new insights into our present knowledge and understanding of mitochondrial dysfunction in cardiovascular diseases.

Pulmonary arterial hypertension (PAH) is an uncurable vascular remodeling disease of the lung with high mortality and poor prognosis. The pathogenesis has not been investigated fully yet. Mitochondrial dysfunction has been involved in the development of PAH by generating ROS (Bonnet S. et al., 2006; Sutendra. and Michelakis, 2014; Kikuchi N. et al., 2018). In the Research Topic, Zhang and colleagues summarized the alterations of mitochondria during the development of PAH and discussed the involvement of the organelle in the pathogenesis of the disease. It is now well-known that the contractile to synthetic phenotype switching of vascular smooth muscle cells (VSMCs) has been considered a cause of PAH and microgravity-related vascular diseases. One of the most evident alterations during this phenotype switching is mitochondrial remodeling characteristic of decreased mitochondrial biogenesis, increased fission, decreased fusion dynamic, and decreased mitophagy. In this Research Topic, Jiang and colleagues indicated that contractile to synthetic phenotype switching of VSMCs and concomitant mitochondrial remodeling is dependent on the loss-of-function of ERα-NRF1-OMI signaling secondary to decreased transcription. Thus, it suggests that the reactivation of the ERα-NRF1-OMI signaling is a potential strategy to treat cerebrovascular remodeling under simulated microgravity. Indeed, the administration of Propyl pyrazole triol, an ERα agonist, ameliorates vascular remodeling. This study supports a well-established epidemiologic survey that the predisposition of females to PAH is more than 1.8 times beyond males and that the use of hormonal therapy in postmenopausal women with PAH is advisable (Frost A. E. et al., 2011; Franco V. et al., 2019). Of note, Jiang and colleagues did not target mitochondrial dysfunction to ameliorate the proliferation of VSMCs.

Mitochondria and Ca^2+^ positively regulate each other (Duchen M. R., 2000). Instant Ca^2+^ entry via opening the mitochondrial calcium uniporter (mtCU) may enhance oxidative metabolism to meet the energy requirement of muscle contraction. Mitochondria are also endowed with the ability to buffer local cellular Ca^2+^ levels during physiological fluctuations of cytosolic Ca^2+^ (Williams G. S. et al., 2013). Numerous stimuli such as electric pulse, elevated extracellular K^+^ levels, and caffeine may enhance cellular Ca^2+^ levels, thereby increasing mitochondria Ca^2+^ entry (Duchen M. R., 2000) (Gherardi G. et al., 2019). In addition, studies have shown that some cell signaling such as estrogen receptor signaling, adrenergic signaling, and insulin signaling may activate mitochondria Ca^2+^ entry (Lobaton C. D. et al., 2005; Gutierrez T. et al., 2014; Jhun B. S. et al., 2018). In the Research Topic, Pablo Sánchez-Aguilera and colleagues reported insulin-like growth factor-1 (IGF-1) as a booster of mitochondrial Ca^2+^ uptake and subsequent oxidative metabolism during cardiomyocyte adaptive growth, which supports the positive inotropic actions of IGF-1 (Ren J. et al., 1999). In addition, IGF-1 signaling can promote mitochondrial biogenesis and mitophagy (Lyons A. et al., 2017). It, therefore, remains intriguing whether mitochondrial Ca^2+^ entry contributes to physiological cardiac hypertrophy induced by persistent activation of IGF-1 signaling. Of note, excessive Ca^2+^ influx occurs in the settings of high and persistent cellular Ca^2+^ environments, which eventually impairs mitochondrial function by multiple mechanisms. In the Research Topic, Liu and colleagues summarized the current knowledge of cytosolic Ca^2+^ overload and mitochondrial homeostasis, while highlighting the interplays between T-tubule, ER, and mitochondria during Ca^2+^ cycling in cardiomyocytes. When excessive Ca^2+^ entry occurs, a vicious cycle is established inevitably: mitochondrial Ca^2+^ overload reduces the mitochondrial membrane potential, which potentially induces the generation of mitochondrial reactive oxygen species (mtROS) with superoxide anions as the most abundant species and reduces the rate of mitochondrial energy production, mitochondrial motility, and morphology. Increased mtROS promotes SR calcium leak, which further loads more Ca^2+^ into mitochondria. This vicious cycle causes mitochondrial dysfunction and stress.

MtROS is a concomitant and inevitable event of the electron transport chain during oxidative phosphorylation, which accounts for approximately 90% of cellular ROS (Mailloux R. J., 2020; Tirichen H. et al., 2021). To counteract mtROS, mitochondria have an anti-oxidative system to scavenge ROS. When the mitochondrial anti-oxidative system is impaired, mtROS accumulates and impairs the mitochondrion itself including mitochondrial DNA (mtDNA). In addition, mtROS leaks out to destroy cellular components in the cytosol (Zorov D. B. et al., 2014). MtROS accumulation accompanied by increased 8-oxo-dG content in the mtDNA is frequently detected in aging tissues. MtROS accumulation is, therefore, considered a critical risk factor for aging-associated cardiovascular diseases, and various strategies have been developed to counteract mtROS. One of the most common strategies is the administration of antioxidants that may be transported into mitochondria (Apostolova N. and Victor V. M., 2015). As a critical component of the mitochondrial antioxidative system, glutathione may be transported into mitochondria from the cytosol to counteract mtROS. Therefore, glutathione administration has been used to counteract oxidative stress. In this Research Topic, Nataliіa and colleagues indicated that the administration of glutathione to the aging animal may largely decrease mtROS production and the sensitivity of mPTP to Ca^2+^, to preserve mitochondrial structure, improve cardiac oxygen consumption, restore endothelium-dependent vasorelaxation in a NO-synthase-dependent manner and confers to the heart resistance to ischemia/reperfusion-induced injury. This study illuminates the present view of mtROS as a cause of aging-related cardiovascular diseases. It should be pointed out that the study by Nataliіa and colleagues exhibits the transient efficacy of glutathione administration. Given some clinical trials showing that long-term supplementation of vitamin E, another well-known antioxidant, does not prevent major cardiovascular events and may even increase the risk for heart failure (Eidelman R. S. et al., 2004; Lonn E. et al., 2005), it is intriguing that long-term administration of glutathione persistently prevents mtROS production, thereby protecting against aging-associated cardiovascular diseases and, more attractively, prolonging lifespan.

Mitochondria in endothelial cells occupy only 2%–6% of cytoplasmic volume, much lower than that in other cell types including cardiomyocytes, and generate only less than 20% of cellular ATP. Endothelial mitochondria sense blood oxygen levels and relay this information to cardiomyocytes as well as modulate the vasodilatory response mediated by endothelial nitric oxide. In addition, the opening of mPTP and activation of mitochondrial pathways of apoptosis both result in endothelial cell death. Although relatively lower than that in other cell types, mtROS is a key signaling mediator in endothelial cells. Therefore, mitochondria in endothelial cells are considered a signaling hub, but not a powerhouse. Some articles have reviewed the relationship between mitochondrial dysfunction, endothelial dysfunction, and atherosclerosis (Kirkman D. L. et al., 2021). In this Research Topic, Qu and colleagues focused mainly on mitophagy and atherosclerosis, with updates on elucidating the involvement of endothelial mitochondria in the formation and development of atherosclerosis. However, a comprehensive and solid conclusion that mitophagy is involved in the development of atherosclerosis warrants further studies.

Although mitochondrial dysfunction has been considered a risk factor for various cardiovascular diseases, there are only a few compounds that have been approved so far for the treatment of rare mitochondrial diseases (Singh A. et al., 2021). In the Research Topic, Zhang and colleagues extend the list of compounds that have been clinically tested in the treatment of PAH and related cardiac diseases. Nevertheless, numerous drugs that effectively ameliorate mitochondrial dysfunction *in vitro* and in animals have shown little effect in clinical trials. This is likely attributable to the complexity of primary mitochondrial diseases (PMD) and secondary mitochondrial dysfunctions (SMD). Therefore, the diagnosis of primary causes of cardiovascular diseases might be a prerequisite for targeting mitochondrial dysfunction.

Taken together, the articles on the Research Topic *“Mitochondrial disorders and cardiovascular diseases”* advance our knowledge of mitochondrial dysfunction and strengthen the causal role of mitochondrial dysfunction in the development of cardiovascular diseases. More significantly, the articles highlight the importance to illuminate the mechanisms underlying mitochondrial dysfunction in the setting of cardiovascular diseases, because a limited understanding of the mechanisms of mitochondrial dysfunction currently retards the treatments and preventions of cardiovascular diseases via ameliorating mitochondrial dysfunction. Predictably, once the mechanisms underlying mitochondrial dysfunction are comprehensively illustrated, it will be likely to treat and prevent cardiovascular diseases via targeting mitochondria in the near future.
